# Rabbit IgA Hinges That Resist IgA1 Protease Action Provide Options for Improved IgA-Based Therapeutic Agents

**DOI:** 10.3389/fimmu.2022.907342

**Published:** 2022-06-17

**Authors:** Patrícia de Sousa-Pereira, Dennis K. Lanning, Pedro J. Esteves, Christian Spoerry, Jenny M. Woof, Ana Pinheiro

**Affiliations:** ^1^ CIBIO-UP, Centro de Investigação em Biodiversidade e Recursos Genéticos, Universidade do Porto, InBIO, Laboratório Associado, Campus Agrário de Vairão, Vairão, Portugal; ^2^ BIOPOLIS Program in Genomics, Biodiversity and Land Planning, CIBIO, Campus Agrário de Vairão, Vairão, Portugal; ^3^ School of Life Sciences, University of Dundee, Dundee, United Kingdom; ^4^ Department of Microbiology and Immunology, Stritch School of Medicine, Loyola University Chicago, Maywood, IL, United States; ^5^ CITS—Centro de Investigação em Tecnologias da Saúde, IPSN, CESPU, Gandra, Portugal; ^6^ Department of Microbiology, Tumor and Cell Biology, BioClinicum, Karolinska Institutet, Stockholm, Sweden

**Keywords:** immunoglobulin A, therapeutic antibodies, hinge region, proteases, European rabbit

## Abstract

Immunoglobulin A provides a major line of defence against pathogens and plays a key role in the maintenance of the commensal microbiota in the intestinal tract. Having been shown to be more effective at tumour cell killing than IgG and strongly active against pathogens present in the mucosae, IgA antibodies have been attracting significant attention in recent years for use as therapeutic antibodies. To improve their therapeutic potential, bioengineered IgA forms with increased serum half-life and neutralizing abilities have been developed but the IgA hinge, which impacts susceptibility to bacterial proteases and ability to bridge between target and effector cells, has not yet been explored. The European rabbit has 15 IgA subclasses with exclusive hinge region motifs and varying lengths, constituting a unique model to evaluate the functional capabilities offered by incorporation of longer IgA hinges into immunoglobulins. Hinge regions from rabbit IgAs, featuring different lengths and sequences, were inserted into human IgA1 heavy chain to substitute the IgA1 hinge. These hinges did not appear to affect antigen binding nor the ability of the engineered chimeric IgA1 to bind and trigger FcαRI, as detected by IgA-mediated cell agglutination and release of superoxide by neutrophils. All rabbit hinge-human IgA1 hybrids were resistant to *Clostridrum ramosum* IgA protease enzyme digestion, as predicted by the lack of the cleavage site in the rabbit hinges. Some IgA1s featuring long rabbit hinges were cleaved by *Neisseria meningitidis* IgA1 protease cleavage type 1 or 2 enzymes, despite the lack of the predicted cleavage sites. More interestingly, the hybrid featuring the rabbit IgA15 hinge was not affected by any of the IgA proteases. The IgA15 hinge is longer than that found in human IgA1 and is composed by a unique motif with a stretch of nine consecutive Ser residues. These characteristics allow the preservation of a long hinge, with associated ability to bridge distantly spaced antigens and provide higher avidity binding, while remaining resistant to IgA protease degradation. The data suggest that the rabbit Cα15 hinge represents an interesting alternative hinge sequence for therapeutic human IgA antibodies that remains resistant to proteolytic cleavage.

## Introduction

Immunoglobulin A (IgA) is the most abundant immunoglobulin (Ig) isotype in humans and mammals. Much of it is secreted in mucosal tissues, such as the intestinal and respiratory tract, where it provides a major line of defence against pathogens and plays a key role in the maintenance of the commensal microbiota in the intestinal tract (reviewed in (1). IgA antibodies act by direct neutralisation of various pathogens and their products or by engagement with receptors, such as FcαRI, which trigger different effector functions ranging from anti- to pro- inflammatory responses (2). The characteristics of IgA antibodies and their functions have been attracting significant attention in recent years for use as therapeutic antibodies. These can be administered *via* the oral or nasal pathways, delivering to mucosal organs including the lungs and gastrointestinal tract, with clear benefits over intravenous and subcutaneous routes of delivery, and have been shown to be more effective than IgG in neutralizing several viruses such as HIV, rotavirus, influenza virus and SARS-CoV2, as well as bacterial pathogens most frequently associated with diarrheal diseases (1, 3–7). Design considerations for IgA mAb development have included the limitation of a relatively short serum half-life, and methodologies to aid retention in the protective mucus barrier and gut, and optimization for protease resistance (1, 6). As such, to improve IgA therapeutic potential, bioengineered IgA forms have been developed to increase serum half-life and neutralizing abilities (4, 5, 8) but the IgA hinge has not yet been explored in terms of the functional capabilities it may impart. By exploiting different hinge characteristics, particularly hinge length and composition which will impact on susceptibility to bacterial proteases and ability to bridge between target and effector cells, we may manipulate IgA stability and interactions with its Fc receptors, achieving the enhancement or suppression of IgA functions (2). It is estimated that thousands of novel pathogens circulating in wild mammals are potentially zoonotic. Climate change is driving the cross-species transmission of these pathogens which will reach human populations increasingly in the future (9). The present Covid-19 pandemic is just an example of such an event. With the increased risk for novel important human diseases, it is of utmost importance that the scientific community develops a good toolbox, including diverse and novel strategies for putative mAb therapeutics improvement. The IgA hinge characteristics is one aspect that has been neglected and may bring improvements to IgA therapeutic antibodies.

The IgA1 and IgA2 subclasses of humans and hominoid primates differ considerably in their hinge region composition and length (10). Human IgA1 has an extended hinge, with a duplicated stretch of amino acids, very different from the short, proline rich, human IgA2 hinge (11). The longer hinge of human IgA1 has greater reach, resulting in potential for higher avidity interactions with more distantly spaced antigens, and more efficient bridging between target and effector cells (12). However, this region is susceptible to proteolytic cleavage by several IgA proteases, including post-proline endopeptidases that cleave at either Pro-Ser (type 1 enzymes) or Pro-Thr (type 2 enzymes) peptide bonds within the IgA1 hinge region (13, 14). These proteases are highly specific for the hinge sequence present in human IgA1 and IgA from very closely related humanoid species, but they tend not to cleave the human IgA2 and the IgA of more distantly related mammals, which lack such long hinges (15, 16). On the other hand, a long hinge region is a feature of most rabbit IgA subclasses (17), therefore, they may share the functional advantages of human IgA1 in relation to antigenic reach and target cell bridging through the interaction with their Fc receptor, while remaining resistant to IgA proteases.

The European rabbit (*Oryctolagus cuniculus)* immunoglobulin (Ig) heavy chain genes (IGH) have particularities that set this species apart from all studied mammals, including the presence of multiple IgA subclasses, being the most complex IgA system of all studied mammals (reviewed in (18). Of the 15 IgA subclasses so far identified, at least 11 are expressed and show differential tissue expression (19, 20). Burnett et al. (17) showed that Jackrabbits and Pikas also have several IgA copies and, as such the expansion of the IgA family in the European rabbit should have begun in a Lagomorph ancestor. It has been argued that bacterial IgA proteases may have influenced the duplication of the IGHA genes in lagomorphs, as the different IgA subclasses must show different susceptibilities to proteolytic cleavage (17). The rabbit IgA subclasses present some differences in the constant domains but mostly in the hinge region, for which almost each subclass has its exclusive motif and length (17, 19). The rabbit IgA hinge regions are generally rich in Pro, Ser and Thr residues, but also carry Cys residues, absent in human IgA hinge regions. Rabbit Cα12 is the IgA subclass with the shortest hinge, with only 9 amino acid residues, while most of the subclasses so far identified are composed by 10 to 24 amino acid residues. The hinge from rabbit Cα15 has some interesting features, presenting an extended hinge with a stretch of nine consecutive Ser residues, and no Pro or Thr residues (19). In this study we investigate whether the different sizes of the rabbit IgA hinges considerably affect their ability to bind to antigens and their resistance against proteolytic cleavage by microbial IgA proteases.

The rabbit IgA hinge constitutes a unique model to evaluate the functional capabilities offered by incorporation of longer IgA hinges into Igs. Exploiting different hinge characteristics, particularly hinge length and composition, which will impact resistance to bacterial proteases and ability to bridge between target and effector cells may open up new avenues for development of more effective monoclonal antibodies (mAb) for therapeutic applications.

## Materials and Methods

### Construction of hIgA1/Rabbit Hinge Expression Vectors

A plasmid containing the gene for the heavy chain constant region of human IgA1 downstream of the mouse VNIP gene was used (14, 21). An AgeI restriction site was introduced upstream of the hinge region, i.e. between the CH1 and the CH2 coding sequences, using the Phusion Site-Directed Mutagenesis Kit (Thermo Fisher Scientific) according to manufacturer’s instructions, with the primers Age_mut_fw 5’-TGC CAC CCC CGA CTG TCA CTG CAC CGA CC-3’ and Age_mut_rv 5’-TGG AGT GGA GAG ATG GCC TGA ACC GGT C-3’. The introduction of the AgeI site was confirmed by sequencing.

The plasmid was cut using the restriction enzymes AgeI and XhoI (cutting site downstream of the hinge region) (New England BioLabs) for the removal of the human IgA1 hinge region. The hinges from rabbit Cα4, Cα5, Cα8 (or Cα13), and Cα15, containing the AgeI and XhoI sites at their 5’ and 3’ ends respectively, were synthesised by Integrated DNA Technologies as gBlocks Gene Fragments (supplementary data). Following restriction digestion with AgeI and XhoI, the hinge fragments were inserted into the plasmid. The hinge from rabbit Cα12 was introduced through site-directed mutagenesis using the primers Rb_Ca12_mut_fw 5’-CCC CAT TAC CTG CCA CCC CCG ACT GTC ACT GC-3’ and Rb_Ca12_mut_rv 5’-GGG ATG GAG CAG TCT GGA GTG GAG AGA TGG CCT GAA C-3’. The introduction of the rabbit hinges in place of the human hinge was confirmed in each case by sequencing.

### Preparation and Purification of hIgA1 and hIgA1/Rabbit Hinge Hybrids

The FreeStyle 293 Expression System from Invitrogen was used to produce the hIgA1 and the hIgA1/rabbit hinge hybrids. FreeStyle™ 293-F Cells were kept in GIBCO FreeStyle 293 Expression Medium according to the manufacturer’s instructions. The cells were transfected with an expression vector containing the gene for a mouse anti-NIP λ light chain (21), and an expression vector for either hIgA1 or one of the hinge hybrids, using 293fectin Transfection Reagent. Supernatants were harvested four days after transfection and passed through a 0.45μm filter.

The presence of IgA in the supernatant was confirmed by ELISA. Recombinant antibodies were purified using the CaptureSelect IgA-CH1 (Hu) Affinity Matrix (Thermo Fisher Scientific) according to the manufacturer’s instructions. Purified antibodies were concentrated using Amicon Ultra-15 Centrifugal Filter Units (Merck Millipore Ltd.) and kept in PBS supplemented with 0.1% sodium azide at -20°C.

### Antigen Binding ELISA

96 well MaxiSorp ELISA Nunc plates were coated with 10μg/mL NIP-BSA in coating buffer overnight at 4°C (21). After coating and blocking with 5% (w/v) non-fat milk (Marvel) in PBS-T (Phosphate Buffered Saline + 0.1% Tween), the plate was loaded with different concentrations of hIgA1 and the hinge hybrids for 2 hours at room temperature. An initial antibody concentration of 0.1ug/ul was used followed by dilutions to 1:100, 1:1000 and 1:10000. Antibody detection was performed using goat anti-mouse λ light chain antibody HRP from Bethyl at a dilution of 1:5000, and SureBlue TMB Microwell Peroxidase Substrate, on a Clariostar instrument.

### Digestion of hIgA1 and Hybrids With Microbial IgA Proteases

Protease preparation from *Clostridium ramosum* AK183 supernatant (22) was a kind gift from Knud Poulsen, Aarhus University. Protease preparations from *Neisseria meningitidis* IgA protease cleavage type 1 and type 2 were prepared through precipitation from MC58 (type 1) and 18-172 (type 2) culture supernatants by addition of ammonium sulphate up to 50% saturation and followed by resuspension in Phosphate Buffered saline (PBS) and sterile filtration. 0.1μg of each IgA was incubated with 2μL of protease preparation in a total volume of 20μL (PBS), and incubated overnight at 37°C. The reaction was stopped by adding sample buffer (8 M urea, 1% SDS, 10% glycerol, 4% 2-mercaptoethanol and a trace of bromophenol blue dye in 50 mM Tris-HCl buffer pH 6.8) and boiling for 5 min. The reduced samples were loaded in a 12% polyacrylamide gel, and upon separation, the proteins were transferred to nitrocellulose membranes. The membranes were incubated with goat anti-human IgA (alpha) peroxidase-labelled antibody from SeraCare at a dilution of 1:3000, and developed using Pierce ECL Western Blotting Substrate.

### Rosette Assay

Sheep blood in Alsever’s solution was washed in isotonic borate buffer (pH 8.5). The red blood cells (RBCs) were derivatized in the same buffer containing 10μg/mL of NIP-caproate-0-succinimide for 1 hour at room temperature. After washing with PBS, the RBCs were sensitized with hIgA1 or one of the hinge hybrids at different concentrations (from 200μg/mL to 12.5μg/mL). Coating levels for each antibody were found to be equivalent by reactivity with goat anti-human IgA FITC conjugate from BioRad and assessed by flow cytometry (data not shown). Neutrophils were isolated from healthy volunteers’ blood using the EasySep Direct Human Neutrophil Isolation Kit from StemCell according to the manufacturer’s instructions. 25μL of a 1% suspension of sensitized RBCs and 25μL neutrophils at 2x106 cells/ml in HBSS were incubated with 50μL PBS for 15 min at room temperature, centrifuged for 1 min at 50 x g and further incubated for 90 mins at 37°C. Upon incubation, cells were transferred to a haemocytometer to be counted. A rosette was defined as an effector cell having three or more RBCs attached.

### Chemiluminescence Assay of Respiratory Bursts

Nunc LumiNuc 96-Well plates were coated with 10μg/mL of NIP-BSA in coating buffer and incubated overnight at 4°C. hIgA1 or one of the hinge hybrids were added in triplicate at 50 μg/ml, and the plate incubated overnight at 4°C. After washing with PBS, 100 μl of luminol solution (67μg/ml in HBSS containing 20mM HEPES buffer and 0.1% globulin-free BSA) was added to each well. 50μL of neutrophils (isolated as previously described) at 10^6^ cells/ml in HBSS containing 0.1% BSA were added to each well. The plate was transferred to a plate reader and chemiluminescence was measured at regular intervals for 85min.

## Results

### Binding of hIgA1 and Rabbit Hinge Hybrids to the Antigen

**Figure 1:**
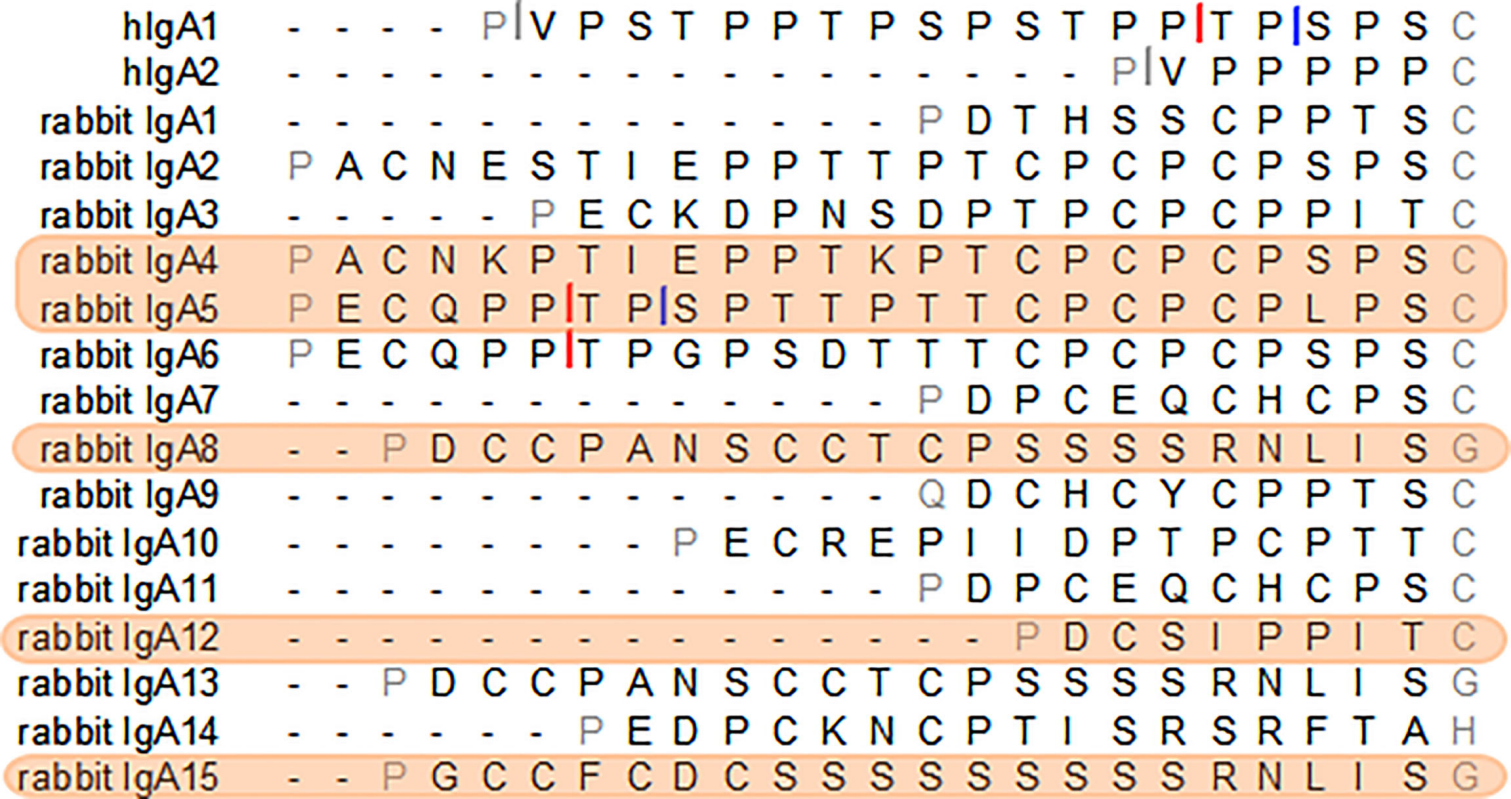
The amino acid sequence of the hinge regions of the human IgA1 and IgA2m (1) and the 15 European rabbit IgAs. Cleavage sites of *Clostridium ramosum* and *Neisseria meningitidis* cleavage type 1 and type 2 proteases in human IgAs are indicated with grey, blue and red bars, respectively. Putative sites of cleavage in the rabbit IgAs are indicated. The selected rabbit IgA hinges for this study are highlighted in orange.

For the purpose of this study, different hinges were chosen due to their characteristics. Our aim was to identify long hinges with improved characteristics. The Cα12 hinge was selected to test the impact of a very short hinge. From the longest hinges, two have clear protease cleavage sites, most do not have apparent IgA protease cleavage sites but possess Pro-Thr or Pro-Ser bonds possibly susceptible to cleavage and one, the hinge from IgA15, has no such bonds. As such, the Cα4 and Cα5 hinges were selected for their extended length (24 amino acids), with the Cα5 hinge containing sequences resembling known IgA protease cleavage sites, while the hinge from Cα4 does not have apparent IgA protease cleavage sites, but possesses Pro-Thr and Pro-Ser bonds possibly susceptible to cleavage. The hinge from Cα8 (or Cα13; these subclasses share the same hinge) was also chosen for sharing the Cα4 characteristics: a long hinge lacking obvious IgA protease cleavage sites but with Pro-Ser bond. The hinge from Cα15, with 22 residues, was chosen for its unique composition (**Figure 1**).

**Figure 2:**
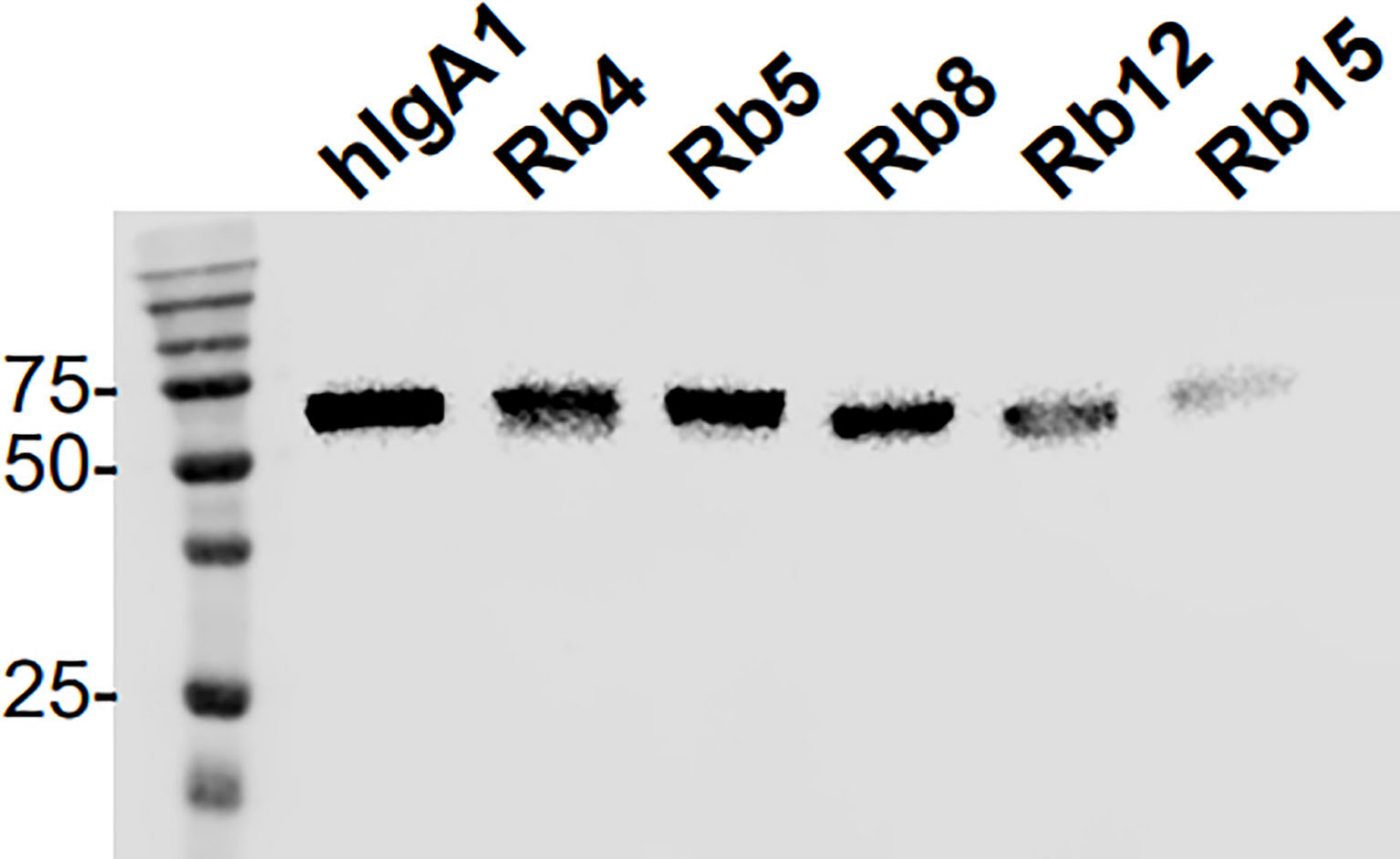
Western blot analysis of hIgA1 and rabbit hinge hybrids separated under reducing conditions and probed with anti-human IgA-peroxidase conjugate. Positions of molecular mass markers in kilodaltons are shown on the left.

Analysis by electrophoresis and immunoblotting under reducing conditions of the hIgA1 and the hIgA1 incorporating the hinges from rabbit IgA subclasses (designated as Rb4, Rb5, Rb8, Rb12 and Rb15, respectively), showed that all antibodies have a similar band at ≈ 60 kDa representing the IgA heavy chain (**Figure 2**). The slight difference in the band position might be attributed to possible differences in glycosylation levels of the heavy chain or due to the different hinge sizes. When compared to hIgA1, Rb4 and Rb5 have four extra amino acid residues in their hinge, Rb8 and Rb15 have two extra amino acid residues, while Rb12 has eleven amino acid residues less. A band at ≈ 25 kDa representing the light chain can equally be detected for all immunoglobulins (data not shown).

**Figure 3:**
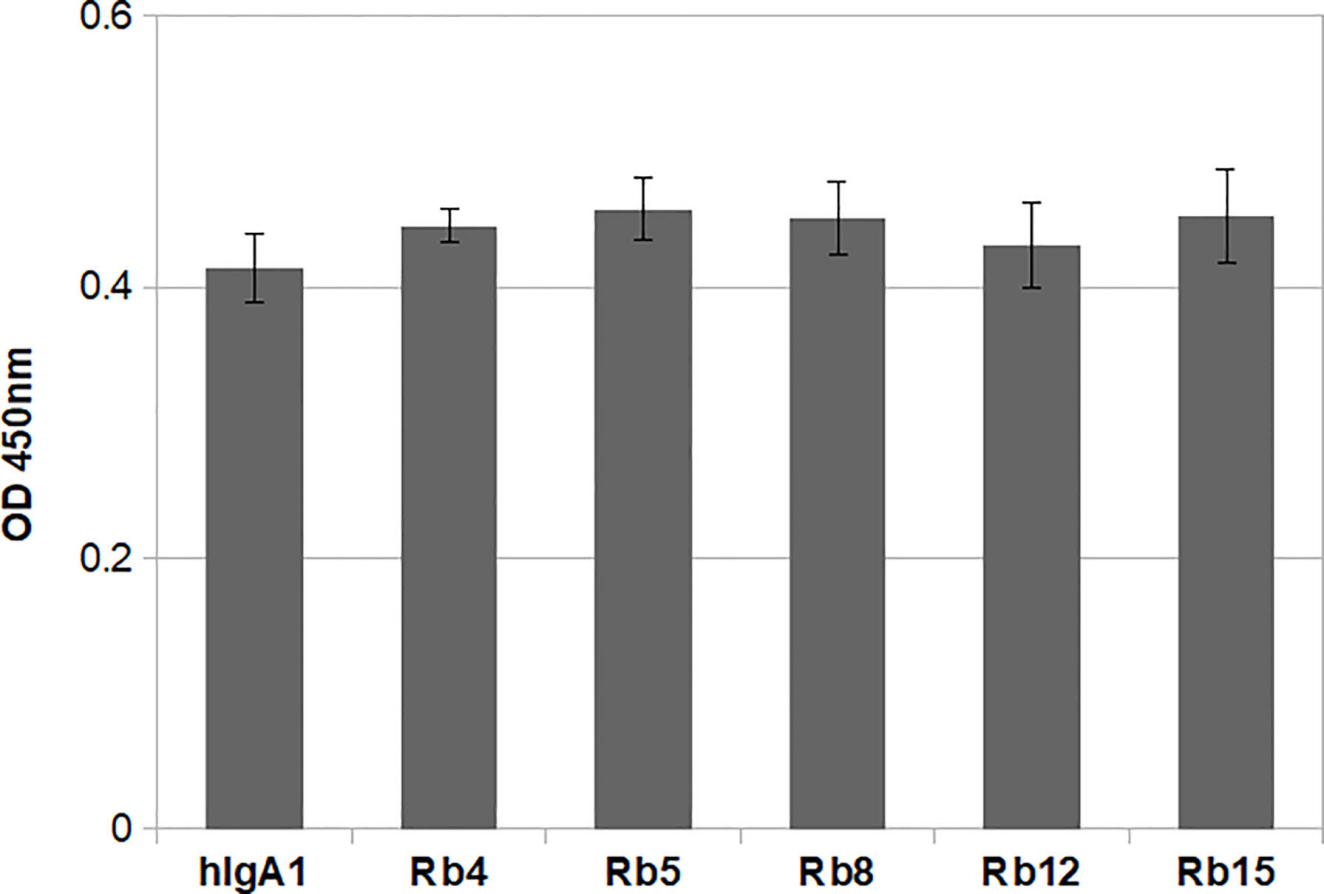
NIP binding ELISA using 0.1μg/mL of each immunoglobulin. The plate was probed with an HRP-conjugated anti-mouse λ light chain antibody. Shown is the mean of triplicate measurements with standard deviation errors. Significance was determined by one-way ANOVA and Tukey’s multiple comparisons test (significant difference among means for p<0.05).

The integrity of the immunoglobulins was also analysed regarding their ability to bind the antigen. All the IgAs were produced containing the heavy and light chain variable regions with specificity for the hapten NIP (3-nitro-4-hydroxy-5-iodophenylacetate). Therefore, binding to the antigen could be evaluated by ELISA using plates coated with NIP. Using equal concentrations of the different immunoglobulins, no significant difference was found regarding antigen binding (**Figure 3**). Besides showing the structural integrity of the immunoglobulins produced, the results also suggest that the difference in hinge size and composition does not significantly affect the ability to bind the antigen.

### Ability of hIgA1 and Rabbit Hinge Hybrids to Bind and Trigger FcαR

**Figure 4:**
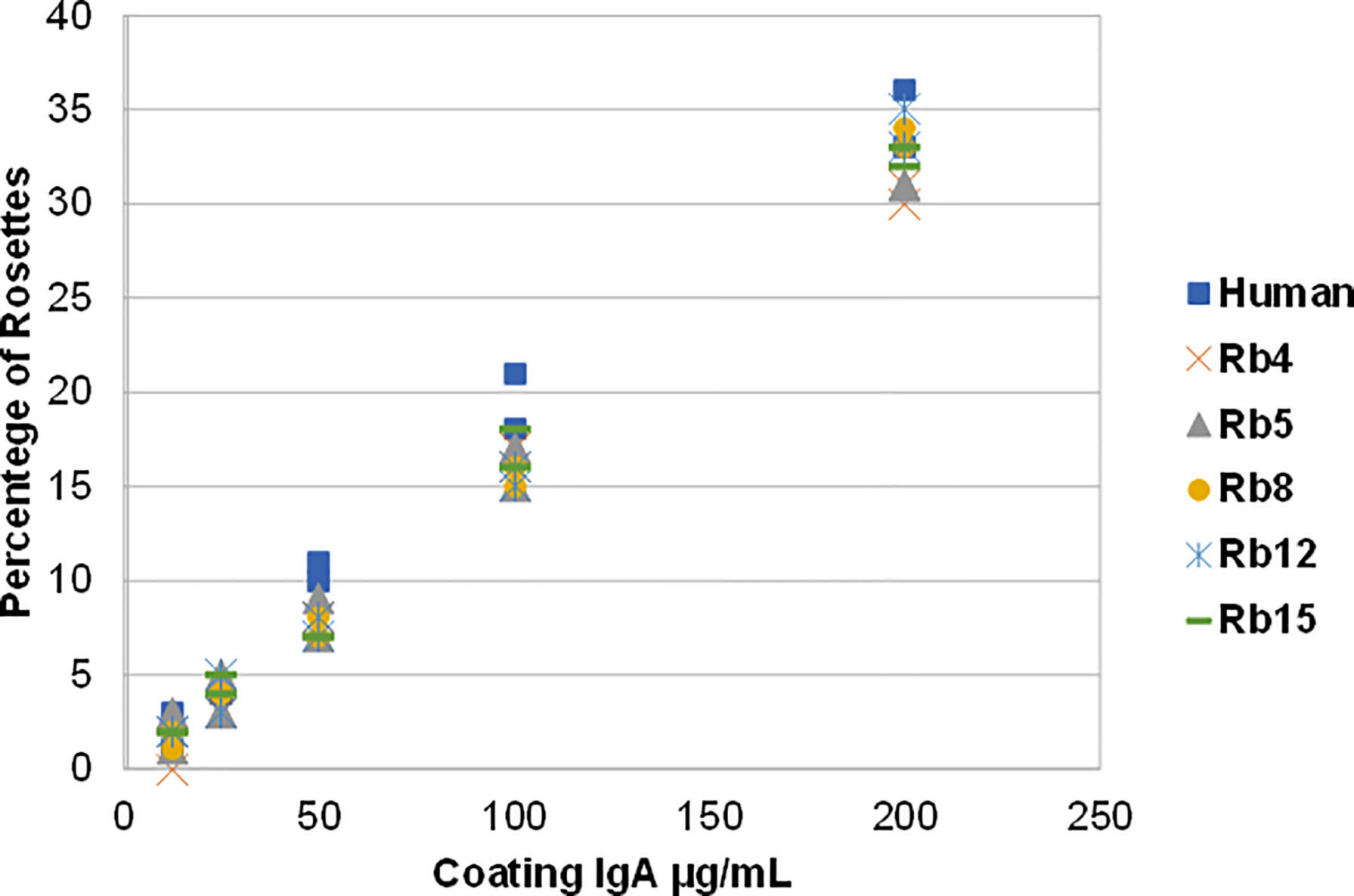
Binding of hIgA1 and rabbit hinge hybrids to human neutrophils assessed by rosette formation. Sheep erythrocytes were coated with different amounts of the immunoglobulins (from 12.5 to 200 μg/mL), and for each condition the percentage of rosettes formed was counted twice (represented in the graph). The experiment was performed twice with the results shown representing a typical experiment.

**Figure 5:**
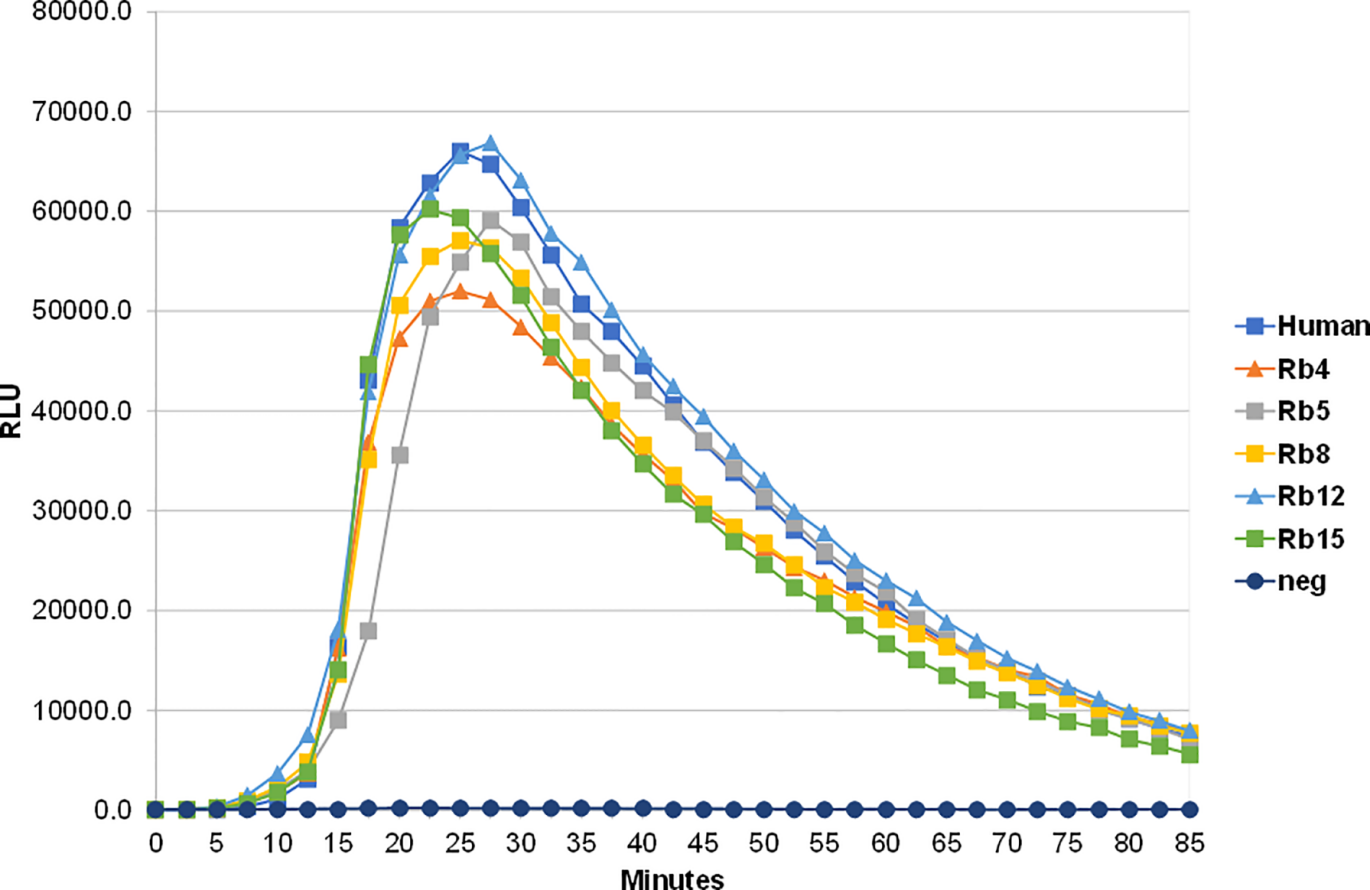
Stimulation of neutrophil-mediated chemiluminescence by hIgA1 and the rabbit hinge hybrids attached to NIP-BSA-coated microtiter plates. Respiratory bursts were induced by the immunoglobulins at 100 μg/ml. A negative control lacking IgA (neg) is also shown. Each point shown is the mean of triplicate determinations. Relative light units (RLU) per second were plotted against time. The experiment was performed twice with the results shown representing a typical experiment.

The ability to interact with the Fcα receptor was evaluated through rosette formation between NIP-coated erythrocytes and neutrophils that naturally express this receptor. All immunoglobulins showed a comparable ability to mediate rosette formation, with an increased percentage of rosette formation observed for the erythrocytes coated with higher concentrations of antibody (**Figure 4**). In addition to rosette formation, engaging of the Fcα receptor was also evaluated by the triggering of a respiratory burst. Neutrophils were added to plates coated with equal amounts of the different immunoglobulins, allowing cross-linking of the Fcα receptor by IgA, causing the release of reactive oxygen species, such as superoxide, which could be measured by a chemiluminescence assay. This assay showed that all the rabbit hinge hybrids produced a comparable respiratory burst to that seen for the hIgA1, once more reaffirming that the replacement of the human IgA1 hinge by those of rabbit IgA does not affect the effector functions of the antibody (**Figure 5**).

### Effect of Different Microbial IgA Proteases in hIgA1 and the Rabbit Hinge Hybrids

**Figure 6:**
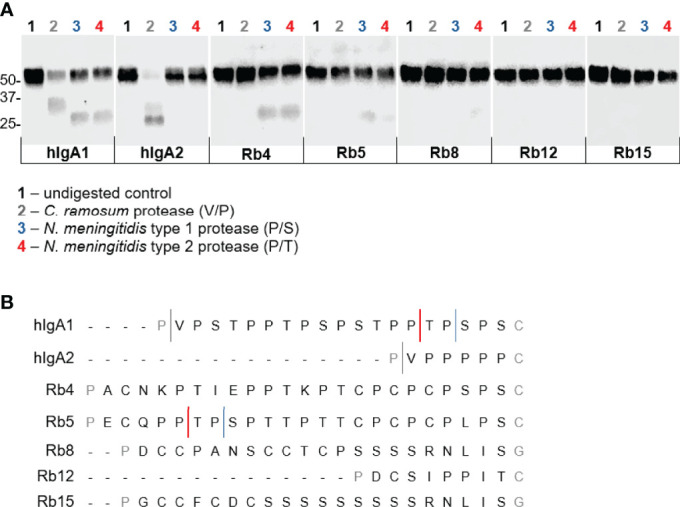
Action of IgA proteases derived from *Clostridium ramosum* and *Neisseria meningitidis* on hIgA1, IgA2m (1) and rabbit hinge hybrids. **(A)** Western blot analysis of proteins separated under reducing conditions and probed with anti-human IgA-peroxidase conjugate. Positions of molecular mass markers are shown in kilodaltons on the left. **(B)** Representation of the amino acid sequence of the human IgA1 and IgA2m (1) hinge region and the cleavage sites of different bacterial IgA proteases. Rabbit Cα4, Cα5, Cα8 (or Cα13), Cα12 and Cα15 hinge regions are also represented, and putative cleavage sites are depicted.

Enzyme preparations from *Clostridium ramosum* and *Neisseria meningitidis* strains producing a cleavage type 1 and cleavage type 2 proteases were used to digest the different immunoglobulins. As expected, the three enzyme preparations cleaved hIgA1 heavy chain in the hinge region to generate Fab and Fc fragments, proving their protease activity (**Figure 6A**). The *C. ramosum* enzyme, through the recognition of the cutting site Pro-Val present at the beginning of the human IgA hinges, was therefore also able to cleave hIgA2m (1). The lack of this cutting site in the studied rabbit hinges presumably rendered the rabbit hinge hybrids resistant to *C. ramosum* enzyme digestion.

The *N. meningitidis* cleavage type 1 and type 2 IgA proteases are known to recognize specific cleavage sites in the hIgA1 hinge (**Figure 6B**). Similar post-proline sites can be found in the rabbit hinge hybrid Rb5, which were found to be cleaved by both *N. meningitidis* type 1 and type 2 enzymes, although, the lower bands corresponding to the Fc region were not as strong as the corresponding band found with hIgA1, suggesting an incomplete digestion. Although the usual Pro-Ser cutting site for the *N. meningitidis* type 1 protease is not present in any other rabbit hinge hybrids, the Rb4 immunoglobulin was unexpectedly also cleaved by this enzyme. The position of the cleavage site is not clear. The *N. meningitidis* type 2 protease also cleaves Rb4 and to a lesser extent Rb8 (**Figure 6A**, notice the faint band), despite the lack of the predicted cleavage site in each case. The bands detected were often faint, but they were detected in all experiments, and their size suggests a specific cleavage in the hinge region. Rb12, the rabbit hinge hybrid with the shortest hinge, was not cleaved by any of the proteases studied here. More interestingly, Rb15, with a hinge longer than that found in hIgA1, was also not affected by any of the proteases.

## Discussion

The extended hinge region of human IgA1 is associated with an increased flexibility, which allows this subclass to bind bivalently, and therefore with greater avidity, to antigens spaced relatively far apart (23). However, the longer hinge of human IgA1 is associated with susceptibility to cleavage by certain bacterial proteases. These are produced by some important respiratory and genital tract pathogens, such as *Streptococcus pneumoniae*, *Haemophilus influenzae*, *Neisseria meningitidis* and *Neisseria gonorrhoeae*. The IgA1 proteases that they produce specifically cleave human IgA1 in the hinge region, generating Fab and Fc fragments, splitting the immunoglobulin’s antigen-recognition capability away from its effector function. Doing so, these pathogens evade IgA1-mediated elimination mechanisms, remaining free to initiate infection at mucosal sites (24, 25). Therefore, the creation of a long hinge with inherent resistance to IgA proteases could present an advantage for creating new therapeutic antibodies.

So far, 15 different IgA subclasses have been identified in the domestic rabbit, comprising a variety of hinge compositions and lengths. The hinge region in rabbit IgA can vary from 9 amino acid residues (Cα12) to 24 amino acid residues (Cα2, Cα4, Cα5 and Cα6). The Cα15 hinge has a very different amino acid composition than other hinges, being composed of 22 amino acid residues, 9 of which form a serine stretch (17, 19). Due to their characteristics, the hinges from Cα4, Cα5, Cα8, Cα12 and Cα15 were chosen for this study and inserted into the hIgA1 heavy chain (designated as Rb4, Rb5, Rb8, Rb12 and Rb15, respectively). The different lengths of these hinges did not appear to affect antigen binding (**Figure 3**), but the greater flexibility of human IgA1 is thought to be advantageous for binding epitopes that are spatially widely separated (23). Therefore, due to the relatively small size of the antigen used, the extra flexibility provided by a longer hinge might not be readily detectable, especially with a direct ELISA. The use of a more sensitive method and possibly a different antigen might further clarify this question.

As expected, the replacement of the human IgA1 hinge with those of different rabbit IgA subclasses did not affect the ability of the antibody to bind to FcαRI. Binding to this receptor occurs through the Fc region (26), which was not modified in this study. Therefore, the differences in hinge size and composition did not affect the ability to bind and trigger FcαRI, as detected by IgA-mediated rosetting (cell agglutination) assay and release of superoxide by neutrophils (**Figures 3**, **4**).

Some of the hinges used in this study are of similar length or even longer than the human IgA1 hinge, which is recognized and cleaved by different bacterial proteases. Human IgA1 was shown to be susceptible to IgA1 zinc metalloproteases from different streptococcal species, as well as to IgA1 serine proteases present in *Haemophilus* and *Neisseria* species (27). A protease found in *C. ramosum* is so far the only one shown to cleave not only human IgA1, but also the human IgA2m (1) allotype. This IgA protease cleaves at the Pro-Val site located in the upper hinge region of hIgA1 and hIgA2m (1), and is not able to cleave the IgA2m (2) allotype due to a Pro->Arg substitution at this position (28).

In this study, the rabbit hinge hybrids were incubated with the IgA proteases from *C. ramosum*, and type 1 and type 2 enzymes from *N. meningitidis*. As expected, the IgA protease from *C. ramosum* was able to cleave human IgA1 and human IgA2m (1) allotype, but were ineffective against the different rabbit hinges that lack any Pro-Val sites (**Figure 5**). The type 1 IgA protease present in *N. meningitidis* is known to cleave at Pro-Ser peptide bonds, while the type 2 cuts at Pro-Thr sites (29). Although there are multiple Pro-Ser and Pro-Thr sites in human IgA1, these enzymes appear to be very specific and cleave at the positions located in the C-terminal portion of the hinge (**Figure 6B**). Potential cleavage sites for *N. meningitidis* IgA1 proteases are also found in Rb5 in a motif very similar to that found in human IgA1, thus, as expected, Rb5 is cleaved by both proteases. This hinge has several other Pro-Thr sites and an alternative Pro-Ser position, allowing for the possibility of other cutting sites. Rb4 hinge has several Pro-Thr sites throughout the hinge, and two possible Pro-Ser cleavage sites at the C-terminal region, and is indeed cut by both type 1 and type 2 proteases. The data provided, however, do not allow identification of the sites used for cleavage. Most curiously, the hinge from Rb8 has a unique Pro-Ser site, however it appeared to be cleaved by the type 2 protease (Pro-Thr specific) and not the type 1 protease (Pro-Ser specific) (**Figure 6A**, a faint band was observed in all experiments).

The results do not come totally unexpected, since it was previously shown that IgA proteases can cleave at the hinge region even when the specific cutting site is not present. Type 1 proteases from *Neisseria* and *Haemophilus* spp were shown to be able to cleave hinge segments that lack any Pro-Ser, and instead, *Haemophilus influenzae* was shown to cleave Pro-Thr peptide bonds close to the N-terminal region. Similarly, type 2 IgA proteases were shown to cut hinges that do not have any Pro-Thr sites (30). Moreover, *Neisseria meningitidis* cleavage type 1 IgA1 protease has been demonstrated to cleave specifically human IgG3, despite all IgG subtypes have the identified Pro-Ala cleavage motif in the lower hinge region in common (31). Therefore, it remains difficult to predict where these IgA proteases cleave the hinge region purely based on their amino acid sequence.

It is also important to note that the cleavage of Rb8 by *N. meningitidis* type 2 protease is very weak, and the cleavage of Rb5 by both type 1 and type 2 enzymes appears also to be weaker when compared to Rb4 or the human IgA1. This may be related to the position of the available cutting sites, since it was proposed that the cleavage efficiency is dependent on the position of the cleavable peptide bonds. Indeed, it was shown that *Neisseria* and *Haemophilus* spp proteases require some sort of spacer at either the N- or C-terminal region of the hinge for successfully access to the cutting site (27).

It was previously shown that the Cys present immediately downstream of the hinge region in human IgA forms a disulphide bridge with a Cys on the other heavy chain, which can restrict movement of the two chains relative to each other around this region. Hence, it was suggested that one heavy chain may be sterically obstructed by the other heavy chain in this region, limiting the access of the IgA proteases to their cutting sites (27). The presence of several Cys residues in the rabbit hinges may enable the creation of disulphide bonds with their counterparts, which, by obstructing the hinge region of both heavy chains, could block the access of IgA proteases. However, as the results show that rabbit hinges containing Cys residues can still be cut by different IgA proteases, it remains to be determined if these Cys residues can indeed create interchain disulphide bonds.

The reduced length and the lack of any predictable cutting site in the Rb12 hinge render this region resistant to IgA proteases, but most interestingly, the Rb15 hinge is also not cut by any of the proteases used in this study. Despite being relatively long, this hinge does not have any Pro-Thr or Pro-Ser sites, but contains a stretch of 9 consecutive serines that was suggested to serve as a spacer between the Fab and Fc regions (19). Ser, a small and slightly polar amino acid, is commonly found in tight turns on the protein surface where, similarly to Pro, it can form a hydrogen bond with the protein backbone (32). Ser residues can also carry O-glycans. These can affect the hinge flexibility, protect the extended hinge regions from bacterial proteases and can further bind pathogens (33). Therefore, the characteristics of Rb15 hinge would allow the preservation of a long hinge, with 22 residues, while remaining resistant to IgA protease degradation.

The effect of streptococcal IgA proteases was not evaluated in this study. These proteases recognize the Pro-Thr site located at the N-terminal region of the human IgA1 hinge. However, using hinge mutants it was shown that some of these proteases can cut even in the absence of the Pro-Thr motif (30). The Rb15 hinge does not have any Pro residues, which seem to be involved in the cutting site of several IgA proteases, suggesting some level of protection against these proteases.

The data suggests that the rabbit Cα15 hinge can be an interesting alternative for a long hinge that remains resistant to IgA protease cleavage, raising new possibilities for therapeutic IgA antibodies engineered with improved functions, an area of increasing biotechnological interest (7). This hinge allows binding with equal efficiency to small antigens, and does not affect the Fc-mediated effector function, while remaining resistant to some IgA proteases. Nonetheless, the effect of the serine stretch on the structure of this hinge and whether its composition allows for a hinge flexibility similar to that in human IgA1 remains unknown and can only be determined by specific structural studies.

## Data Availability Statement

The raw data supporting the conclusions of this article will be made available by the authors, without undue reservation.

## Author Contributions

PS-P and CS performed the laboratory experiments. PS-P and AP wrote the manuscript. PS-P, AP, DL, PE, CS and JW discussed the data. JW and AP conceived the study. All authors edited the manuscript and approved the final draft. All authors contributed to the article and approved the submitted version.

## Funding

This work was co-funded via national funds through FCT—Foundation for Science and Technology and EU funds European Regional Development Fund (ERDF) under the project PTDC/BIA-OUT/29667/2017 – POCI-01-0145-FEDER-029667 FCT also supported the post-doctoral fellowships of AP (ref. SFRH/BPD/117451/2016) and the FCT Investigator grant of PJE (CEECIND/CP1601/CT0005).

## Conflict of Interest

The authors declare that the research was conducted in the absence of any commercial or financial relationships that could be construed as a potential conflict of interest.

## Publisher’s Note

All claims expressed in this article are solely those of the authors and do not necessarily represent those of their affiliated organizations, or those of the publisher, the editors and the reviewers. Any product that may be evaluated in this article, or claim that may be made by its manufacturer, is not guaranteed or endorsed by the publisher.
